# Remote Phonon Scattering in Two-Dimensional InSe FETs with High-*κ* Gate Stack

**DOI:** 10.3390/mi9120674

**Published:** 2018-12-19

**Authors:** Pengying Chang, Xiaoyan Liu, Fei Liu, Gang Du

**Affiliations:** Institute of Microelectronics, Peking University, Beijing 100871, China; fliu003@gmail.com (F.L.); gangdu@pku.edu.cn (G.D.)

**Keywords:** two-dimensional material, field effect transistor, indium selenide, phonon scattering, mobility, high-*κ* dielectric

## Abstract

This work focuses on the effect of remote phonon arising from the substrate and high-*κ* gate dielectric on electron mobility in two-dimensional (2D) InSe field-effect transistors (FETs). The electrostatic characteristic under quantum confinement is derived by self-consistently solving the Poisson and Schrödinger equations using the effective mass approximation. Then mobility is calculated by the Kubo–Greenwood formula accounting for the remote phonon scattering (RPS) as well as the intrinsic phonon scatterings, including the acoustic phonon, homopolar phonon, optical phonon scatterings, and Fröhlich interaction. Using the above method, the mobility degradation due to remote phonon is comprehensively explored in single- and dual-gate InSe FETs utilizing SiO_2_, Al_2_O_3_, and HfO_2_ as gate dielectric respectively. We unveil the origin of temperature, inversion density, and thickness dependence of carrier mobility. Simulations indicate that remote phonon and Fröhlich interaction plays a comparatively major role in determining the electron transport in InSe. Mobility is more severely degraded by remote phonon of HfO_2_ dielectric than Al_2_O_3_ and SiO_2_ dielectric, which can be effectively insulated by introducing a SiO_2_ interfacial layer between the high-*κ* dielectric and InSe. Due to its smaller in-plane and quantization effective masses, mobility begins to increase at higher density as carriers become degenerate, and mobility degradation with a reduced layer number is much stronger in InSe compared with MoS_2_.

## 1. Introduction

The compelling demand for higher performance and lower power consumption in complementary metal-oxide-semiconductor (CMOS) field-effect transistors (FETs) has highlighted the quest for devices and architectures based on new materials [1]. Performance boosters such as strain, high-*κ* dielectric, metal gate, and three-dimensional (3D) devices have enabled extraordinary improvement of performance in the past 60 years [2,3]. Recently, two-dimensional (2D) van der Waals semiconductors hold great potential for optics and electronics application due to their unique properties, including the atomic thickness, tunable bandgap, and dangling-bond-free surface, which achieves improved gate control over the channel and reduced short channel effects [4,5]. So far, many classes of 2D material-based devices have been extensively studied, such as graphene, transition metal dichalcogenides (TMDs), and black phosphorus [6,7,8]. Very recently, few-layer InSe has attracted much attention due to its highly promising prospect as channel material for FETs, offering small effective mass of electron ~0.14 *m*_0_ and high electron mobility up to ~10^3^ cm^2^/Vs at room temperature obtained by experimental measurements [9,10,11]. Therefore, InSe has advantages of a similar gap as silicon, 2D nature as graphene, higher mobility than TMDs, and higher environmental stability than black phosphorus. In addition, electrostatic tunability of spin-orbit coupling in InSe has been identified, showing potential in devising III-VI based spintronic devices [12,13].

However, the charge transport properties in InSe FET have not been well understood and starve for comprehensive investigation. More recently, the ballistic performance of mono- and multi-layer InSe FET is studied by the first-principles calculation and the top of the barrier model [14], and temperature-dependent phonon-limited mobility is estimated by the physical modeling of intrinsic scattering mechanisms [15]. On the other hand, charge transport behavior is very sensitive to external surroundings, such as gaseous adsorbates from air and trapped charges in substrates [16], and their electronic performance is generally lower than their intrinsic values. Previous studies of back-gated multilayer InSe FET on various substrates (bare SiO_2_, bare Al_2_O_3_, poly(methyl methacrylate) (PMMA)/SiO_2_, and PMMA/Al_2_O_3_) have reported the carrier mobility ranging from 2.2 cm^2^/Vs to 1055 cm^2^/Vs at low operating voltage [10], while dual-gated InSe FET on hexagonal boron nitride (hBN)/SiO_2_ show an excellent mobility approaching 10^3^ cm^2^/Vs and 10^4^ cm^2^/Vs at room and liquid-helium temperatures respectively [9]. It is apparently suggested that the introduction of substrate and gate dielectric has a strong effect on the electron mobility, which can be generally contributed to the extrinsic scatterings from surface roughness (SRS), interfacial Coulomb impurities (CIS), and remote phonon scatterings (RPS) [17,18]. Atomic flatness of 2D materials makes them immune to SR scattering, while CIS can be lowered or eliminated as possible by improving the fabrication process. Therefore, only remote phonon can be regarded as an intrinsic factor arising from the dielectric environment, and open questions remain as to its role in determining the electron transport in atomically-thin InSe FETs.

In this paper, the effect of remote phonons arising from the substrate and high-*κ* dielectric together with the intrinsic phonons of the InSe channel on electron transport is studied based on the physical modeling by self-consistently solving the Poisson and Schrödinger equations and employing the Kubo–Greenwood formula. Mobility behaviors in single-gate and dual-gate InSe FET with various gate dielectric are theoretically explored and analyzed as a function of temperature, inversion density, InSe layer number, and SiO_2_ interfacial layer thickness. Acoustic phonons and optical phonons—as well as homopolar phonons—have a minor effect on electron mobility, while remote phonons and Fröhlich interaction play a comparatively major role in determining the electron transport in InSe. Compared with MoS_2_, much smaller effective masses of electron in InSe give rise to a great enhancement of mobility at high density as carriers become degenerate. Simulation results in this work provide physical insight into the mobility behavior of InSe FET for carrier mobility optimization from the theoretical viewpoint.

This paper is organized as follows. Section 2 describes the device structures and simulation methods, especially the physical models of remote phonon depending on the gate stack. In Section 3, we present simulation results of mobility and corresponding explanations. Finally, the conclusion is drawn in Section 4.

## 2. Device Structures and Simulation Methods

**Figure 1:**
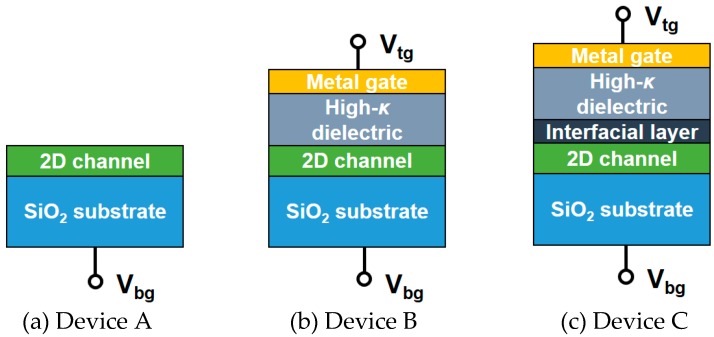
Simulated device structure with atomically thin InSe channel in this work. (**a**) Back-gate InSe field-effect transistors (FET) with SiO_2_ substrate as gate dielectric. (**b**) Dual-gate InSe FET with high-*κ* dielectric as top-gate dielectric and SiO_2_ substrate as back-gate dielectric. (**c**) The same structure as (**b**) with additional SiO_2_ interfacial layer embedded between the InSe channel and high-*κ* dielectric. For the dual-gate structure, high-*κ* dielectric is covered with a metal gate.

**Figure 2:**
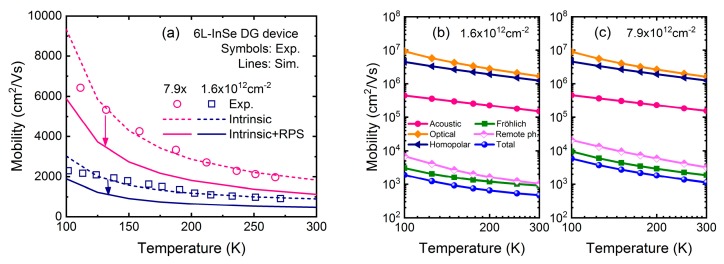
Temperature-dependent mobility in six-layer InSe FETs at inversion density of 1.6 × 10^12^ cm^−2^ and 7.9 × 10^12^ cm^−2^, respectively. (**a**) Comparison between the experimental mobility (symbols) and calculated intrinsic mobility without remote phonon scattering (RPS) (dashed lines), which shows an excellent agreement. In contrast, the mobility with RPS (solid lines) is significantly degraded. (**b**,**c**) Contributions of each scattering mechanisms to the total mobility for different inversion density respectively. Intrinsic scatterings include acoustic (AC), optical (OP), and homopolar (HO) phonon scatterings as well as Fröhlich interaction, while extrinsic scattering is remote phonon scattering arising from the HfO_2_ high-*κ* dielectric.

Simulated device structures with 2D-layered InSe channel are shown in Figure 1, where the intrinsic channel without doping is assumed. Figure 1a shows the back-gate (single-gate) InSe FET with SiO_2_ substrate as gate dielectric. Figure 1b shows the top-gate (dual-gate) InSe FETs with high-*κ* dielectric as top dielectric and SiO_2_ substrate as back dielectric. Figure 1c shows the structure with additional SiO_2_ interfacial layer (ITL) embedded between the InSe channel and high-*κ* dielectric compared with Figure 1b. In the case of single-gate devices, only the back gate is biased with V_bg_, while the back gate is grounded and the top gate is biased with V_tg_ for the dual-gated devices. In this work, traditionally used high-*κ* dielectrics of HfO_2_ and Al_2_O_3_ are comprehensively studied, with corresponding parameters listed in Table 1. Except for Figure 2, all the simulation results are calculated at room temperature (300 K).

We start the calculation by obtaining the electrostatic characteristic of the two-dimensional electron gas (2DEG) in InSe layer by self-consistently solving the Poisson and Schrödinger equations using the effective mass approximation with nonparabolicity correction, inherently accounting for the quantum confinement effects [19]. Particularly the energy dispersion of 2D-layered InSe is described by the thickness-dependent effective masses obtained from first-principles calculation, as shown in our previous work [14]. Next, the matrix elements and the scattering rates are calculated through the Fermi golden rule [19]. Physical models for electron mobility include the remote phonon scattering (RPS) arising from the high-*κ* dielectric as well as the intrinsic phonon scatterings of channel material, including the acoustic (AC) phonon-, homopolar (HO) phonon-, optical (OP) phonon- scatterings, and Fröhlich interaction (POP) [20,21,22,23]. For AC phonons, elastic and isotropic approximations are adopted. The HO and OP scatterings are treated as inelastic and isotropic process. For POP and RP scatterings, inelastic and anisotropic characteristic are considered. Once the scattering rates are obtained, the mobility is calculated by the Kubo–Greenwood formula employing the momentum relaxation time approximation. The parameters for mobility calculation in few-layer InSe are taken from our previous work [15].

For remote phonon induced by SiO_2_ substrate, the semi-infinite substrate is assumed, and the dispersion relationship for the remote phonon model can be written as [19]

(1) $$\omega_{RP}=\omega_{TO1,{\mathrm{SiO}}_{2}}\sqrt{\frac{\varepsilon_{\mathrm{InSe}}+\varepsilon_{{\mathrm{SiO}}_{2},0}}{\varepsilon_{\mathrm{InSe}}+\varepsilon_{{\mathrm{SiO}}_{2},\infty }}}$$

where *ω*_*TO*1,SiO2_ is the low-frequency phonon mode of SiO_2_, *ε*_InSe_ is the dielectric constant of InSe. The potential amplitude of the remote phonon is written as

(2) $$\frac{1}{{\hat{\varepsilon }}_{RP}}=\frac{1}{\varepsilon_{\mathrm{InSe}}+\varepsilon_{{\mathrm{SiO}}_{2},\infty }}-\frac{1}{\varepsilon_{\mathrm{InSe}}+\varepsilon_{{\mathrm{SiO}}_{2},0}}$$

For the remote phonon induced by top gate dielectric, high-*κ* dielectric covered with a metal gate is employed in the simulation. As shown in Table 1, the frequencies of two polar phonons in high-*κ* dielectrics such Al_2_O_3_ and HfO_2_ show great discrepancy. Hence, for simplicity, only the low-frequency phonon mode in high-*κ* gate dielectric is considered [19]. For high-*κ* dielectric with a metal gate as shown in Figure 1b, the dispersion relationship is

(3) $$\omega_{RP}=\omega_{TO1,HK}\frac{{\left(\varepsilon_{\mathrm{InSe}}\left(\frac{1-e^{-2qT_{HK}}}{1+e^{-2qT_{HK}}}\right)+\varepsilon_{HK,0}\right)}^{1/2}}{{\left(\varepsilon_{\mathrm{InSe}}\left(\frac{1-e^{-2qT_{HK}}}{1+e^{-2qT_{HK}}}\right)+\varepsilon_{HK,\mathrm{int}}\right)}^{1/2}}$$

where *ω*_*TO*1,*HK*_ is the low-frequency phonon mode of high-*κ* dielectric, *ε*_*HK*,0_ and *ε_HK,int_* are the dielectric constant at the static and intermediate frequency, *T_HK_* is the thickness of top gate dielectric, and *q* = |*k* − *k*′| is the remote phonon momentum. The effective dielectric constant depending on the frequency dependent dielectric constant of the high-*κ* material can be written as

(4) $$\varepsilon_{eff}=\varepsilon_{HK}\left(\omega \right)\frac{1+e^{-2qT_{HK}}}{1-e^{-2qT_{HK}}}+\varepsilon_{\mathrm{InSe}}$$

and then the corresponding potential amplitude is

(5) $$\frac{1}{{\hat{\varepsilon }}_{RP}}=\frac{1}{\varepsilon_{eff}(\varepsilon_{HK,\mathrm{int}})}-\frac{1}{\varepsilon_{eff}(\varepsilon_{HK,0})}$$

For the high-*κ* gate stack with a SiO_2_ interfacial layer, namely ITL/high-*κ*/metal-gate stack as shown in Figure 1c, the dispersion relationship is [16]

(6) $$\begin{array}{l} \omega_{RP}=\omega_{TO1,HK}{\left[\varepsilon_{{\mathrm{SiO}}_{2},0}\left(\frac{1-e^{-2qT_{HK}}}{1+e^{-2qT_{HK}}}\right)\left(\frac{1-\frac{\varepsilon_{{\mathrm{SiO}}_{2},0}+\varepsilon_{\mathrm{InSe}}}{\varepsilon_{{\mathrm{SiO}}_{2},0}-\varepsilon_{\mathrm{InSe}}}e^{2qT_{ITL}}}{1+\frac{\varepsilon_{{\mathrm{SiO}}_{2},0}+\varepsilon_{\mathrm{InSe}}}{\varepsilon_{{\mathrm{SiO}}_{2},0}-\varepsilon_{\mathrm{InSe}}}e^{2qT_{ITL}}}\right)-\varepsilon_{HK,0}\right]}^{1/2}\\ \;\times {\left[\varepsilon_{{\mathrm{SiO}}_{2},0}\left(\frac{1-e^{-2qT_{HK}}}{1+e^{-2qT_{HK}}}\right)\left(\frac{1-\frac{\varepsilon_{{\mathrm{SiO}}_{2},0}+\varepsilon_{\mathrm{InSe}}}{\varepsilon_{{\mathrm{SiO}}_{2},0}-\varepsilon_{\mathrm{InSe}}}e^{2qT_{ITL}}}{1+\frac{\varepsilon_{{\mathrm{SiO}}_{2},0}+\varepsilon_{\mathrm{InSe}}}{\varepsilon_{{\mathrm{SiO}}_{2},0}-\varepsilon_{\mathrm{InSe}}}e^{2qT_{ITL}}}\right)-\varepsilon_{HK,\mathrm{int}}\right]}^{-1/2} \end{array}$$

where *T_ITL_* is the thickness of interfacial layer. The effective dielectric constant is

(7) $$\begin{array}{ll} \varepsilon_{eff}\left(\omega \right)= & \varepsilon_{HK}\left(\omega \right)\left[{\left(\frac{\varepsilon_{{\mathrm{SiO}}_{2},0}-\varepsilon_{\mathrm{InSe}}}{2\varepsilon_{{\mathrm{SiO}}_{2},0}}\right)}^{2}e^{-2qT_{ITL}}+{\left(\frac{\varepsilon_{{\mathrm{SiO}}_{2},0}+\varepsilon_{\mathrm{InSe}}}{\varepsilon_{{\mathrm{SiO}}_{2},0}}\right)}^{2}e^{2qT_{ITL}}+2\frac{\varepsilon_{{\mathrm{SiO}}_{2},0}^{2}-\varepsilon_{\mathrm{InSe}}^{2}}{{\left(2\varepsilon_{{\mathrm{SiO}}_{2},0}\right)}^{2}}\right]\cdot \frac{1+e^{-2qT_{HK}}}{1-e^{-2qT_{HK}}}\\ & +\frac{{\left(\varepsilon_{{\mathrm{SiO}}_{2},0}+\varepsilon_{\mathrm{InSe}}\right)}^{2}}{4\varepsilon_{{\mathrm{SiO}}_{2},0}}e^{2qT_{ITL}}-\frac{{\left(\varepsilon_{{\mathrm{SiO}}_{2},0}-\varepsilon_{\mathrm{InSe}}\right)}^{2}}{4\varepsilon_{{\mathrm{SiO}}_{2},0}}e^{-2qT_{ITL}} \end{array}$$

Then the potential amplitude for the ITL/high-*κ*/metal-gate stack can be obtained through the Equations (5) and (7).

## 3. Results and Discussion

To begin with, we calibrate the physical models with the experimental measurement. Figure 2 shows the calculated and experimental temperature-dependent mobility at inversion density of 1.6 × 10^12^ cm^−2^ and 7.9 × 10^12^ cm^−2^ in six-layer InSe dual-gate FET. It should be noted that the experiment results are obtained from the dual-gate InSe FET with channel covered by hexagonal boron nitride (hBN) [9], which insulates InSe from the dielectric environment, leading to the absence of remote phonon scattering. From Figure 2a, considering the intrinsic scatterings by AC, HO, and OP phonon and Fröhlich interaction, the temperature-dependent electron mobility curves measured by Hall effect are reproduced successfully for T > 100 K, where phonon scatterings dominate. The excellent agreement between the simulations and experiments validate our methods and models. It should be pointed out that when temperature is down to 100 K, there is a significant discrepancy of mobility between simulations and experiments due to the fact that Coulomb scattering resulting from the channel impurities and interfacial charges is excluded, which is a dominant factor in determining the carrier mobility in the low-temperature regime.

On the other hand, if high-*κ* dielectric of HfO_2_ is directly deposited on the InSe channel, the mobility is severely degraded from its intrinsic value, as shown in Figure 2a by solid lines. For example, at room temperature, mobility changes from 1808 to 1120 cm^2^/Vs (920 to 464 cm^2^/Vs) at inversion density of 7.9 × 10^12^ cm^−2^ (1.6 × 10^12^ cm^−2^) due to the remote phonon scattering. To understand the mobility behavior in depth, Figure 2b,c plot the contributions of all the considered scattering mechanisms to the total mobility. Compared with AC, OP, and HO phonons, the remote phonon together with Fröhlich interaction plays a comparatively major role in determining the electron transport in InSe FET. This is the objective of this work to focus on the remote phonon scattering in InSe FET with high-*κ* gate stack in the following.

**Figure 3:**
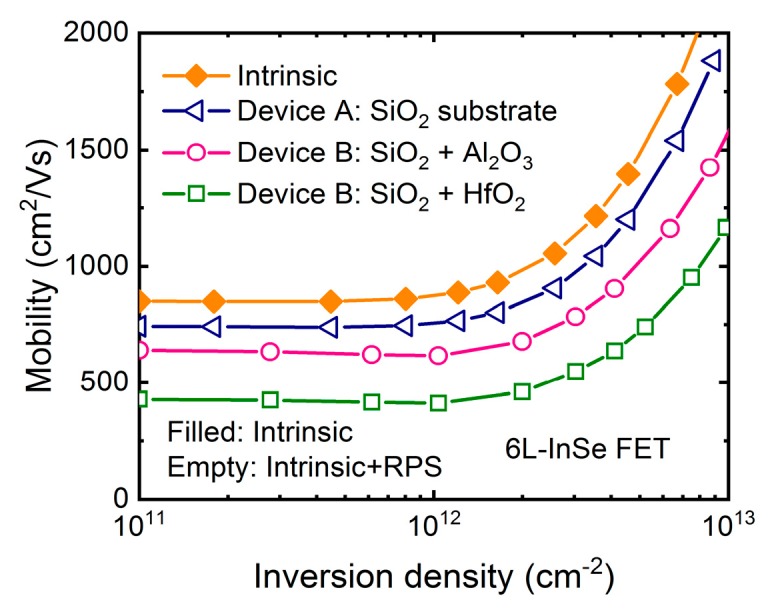
Calculated mobility as a function of inversion density in six-layer InSe FETs with different device structures as shown in Figure 1a,b utilizing Al_2_O_3_ and HfO_2_ as top-gate dielectric and SiO_2_ substrate as back-gate dielectric respectively. Equivalent oxide thickness (EOT) = 1 nm of high-*κ* dielectric is assumed. Filled symbols represent intrinsic phonon-limited mobility for benchmark, while empty symbols represent total mobility including the remote phonon scattering from substrate and high-*κ* dielectric.

The effect of remote phonon originating from the substrate and gate stack on the electron transport of few-layer InSe is shown in Figure 3. The intrinsic phonon-limited mobility in six-layer single-gate InSe FET is ~843 cm^2^/Vs at low inversion density. With SiO_2_ substrate employed, the mobility is degraded to ~735 cm^2^/Vs due to remote phonon. In the case of dual-gate structure, when Al_2_O_3_ and HfO_2_ are used as top-gate dielectric, the additional remote phonon further reduces the mobility to ~634 cm^2^/Vs and 426 cm^2^/Vs respectively. It can be seen that the HfO_2_ dielectric has a much stronger influence of remote phonon than Al_2_O_3_ and SiO_2_ dielectric since it has higher dielectric constant and softer polar vibration mode [20], as listed in Table 1. Particularly, it is worth noting that the smaller permittivity of InSe results in a stronger remote phonon coupling with electrons compared with silicon even in the SiO_2_ case. Despite serious degradation due to remote phonons, the mobility of the six-layer InSe with high-*κ* dielectric is higher than that of silicon on insulator (SOI) device with SiO_2_ dielectric at a comparative thickness [24], revealing its great potential in high-performance logic application.

**Figure 4:**
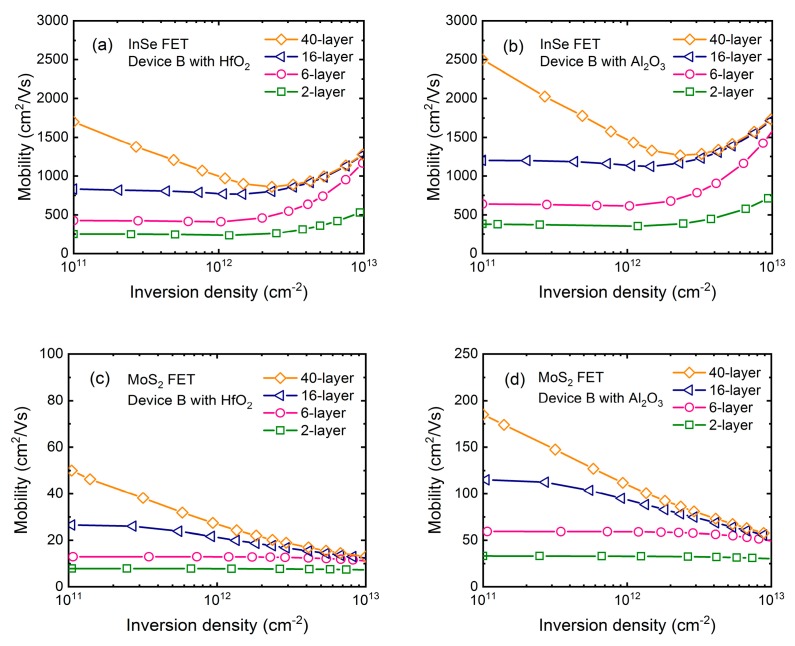
Mobility as a function of inversion density with layer number of 2, 6, 16, and 40 employing the dual-gate structure as shown in Figure 1b in InSe and MoS_2_ FET for comparison. EOT = 1 nm of high-*κ* dielectric is assumed. (**a**) InSe FET with HfO_2_ dielectric. (**b**) InSe FET with Al_2_O_3_ dielectric. (**c**) MoS_2_ FET with HfO_2_ dielectric. (**d**) MoS_2_ FET with Al_2_O_3_.

It is also observed that in six-layer InSe, mobility is increased significantly at higher density, which is against the common sense. To confirm this behavior, mobility in 2-, 6-, 16-, and 40-layer InSe FET is calculated in Figure 4a,b, where HfO_2_ and Al_2_O_3_ dielectric are used separately. At the same time, mobility in MoS_2_ FET using same device structure is also plotted in Figure 4c,d for comparison. In the MoS_2_ case, as inversion density increases, mobility monotonously decreases for thick devices as expected, and remains almost unchanged for thin devices due to strong quantum confinement. In the InSe case, at low density, mobility behavior is consistent with MoS_2_. However, when inversion density is larger than ~2 × 10^12^ cm^−2^, mobility quickly increases regardless of layer number or high-*κ* dielectric. Actually, this is also demonstrated by experimental results in [9] as shown in Figure 2a, which cannot be totally contributed to impurity scattering because at large inversion density and room temperature the screening produced by the inversion layer drastically reduce the Coulomb scattering. Therefore, this exceptional enhancement seems intrinsic for 2D-layered InSe to a great extent.

**Figure 5:**
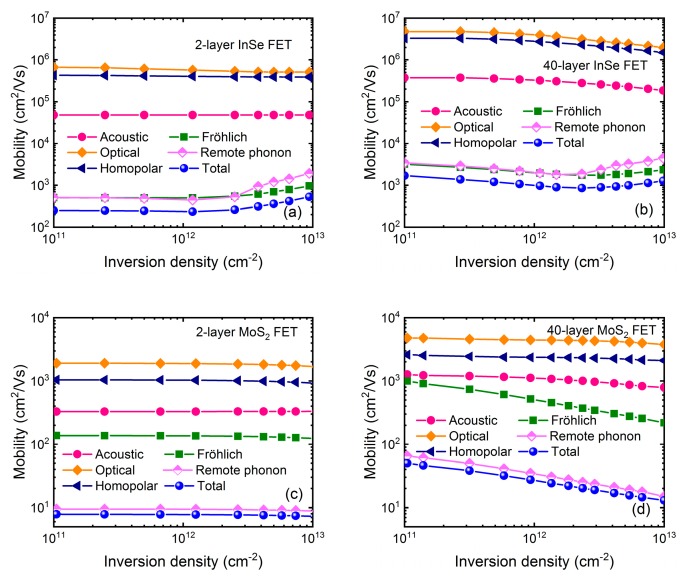
Contributions of each scattering process to the total mobility corresponding to Figure 4a,c for InSe and MoS_2_ FET with HfO_2_ dielectric respectively. (**a**) 2-layer InSe FET. (**b**) 40-layer InSe FET. (**c**) 2-layer MoS_2_ FET. (**d**) 40-layer MoS_2_ FET.

To get physical insight into this mobility behavior, Figure 5a–d shows the contributions of each scattering mechanism to the total mobility in both InSe and MoS_2_ FET with 2- and 40-layer thickness respectively. Consistent with above-mentioned results, mobility behavior is mainly governed by the remote phonon and Fröhlich interaction in all considered devices. We find that their scattering rates increase with inversion density increasing, which should reduce the mobility. In the MoS_2_ case, carriers are always non-degenerate following the Boltzmann distribution, where the mobility is essentially determined by the relaxation times or scattering rates. On the other hand, when inversion density is larger than ~2 × 10^12^ cm^−2^, carriers in InSe FET become degenerate, where subband minimum is lower than Fermi level *E_F_*, and consequently the most influential relaxation times are those for energies close to *E_F_*. Due to their anisotropic property, scattering rates of remote phonon and Fröhlich interaction are much smaller near *E_F_* than those of the subband minimum, giving rise to an enhancement of mobility. This discrepancy between InSe and MoS_2_ FET can be well understood by their effective masses. Firstly, in-plane effective mass of 0.14 *m*_0_ in InSe is much smaller than 0.62 *m*_0_ in MoS_2_ [25] leading to much smaller density-of-states (DOS). In order to obtain the same density, the conduction band minimum is lower than Fermi level over a few *k*_B_*T*, where *k*_B_ is the Boltzmann constant. Secondly, quantization effective mass of 0.08 *m*_0_ in InSe is also much smaller than 0.49 *m*_0_ in MoS_2_ [26]. This is the reason that quantum confinement takes effect in 16-layer InSe, but not until the layer number is reduced to 6 layers in MoS_2_. Actually, less subbands contributing to the carrier transport in InSe need Fermi level being higher to change the density, which makes carriers more degenerate together with the effect of small DOS.

**Figure 6:**
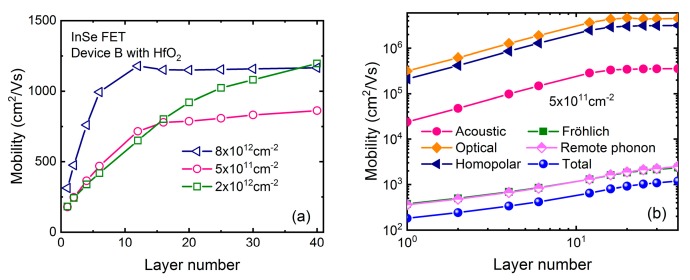
(**a**) Mobility as a function of number of layers ranging from 1 to 40 with different inversion density in InSe FET using HfO_2_ as gate dielectric and SiO_2_ as substrate, as shown in Figure 1b. (**b**) Contributions of each scattering mechanism to the total mobility at low inversion density.

The dependence of mobility on number of layer (NL) in InSe FET is shown in Figure 6a, where inversion densities of 5 × 10^11^, 2 × 10^12^, and 8 × 10^12^ cm^−2^ are considered respectively. In the case of the relatively medium and high density, mobility is almost independent of channel thickness until NL ~15, and then drops rapidly as NL is further reduced. In the low-density case, mobility degradation occurs earlier when NL <40. It is noted that for thicker devices, mobility at 5 × 10^11^ cm^−2^ gradually surpasses the value at 2 × 10^12^ cm^−2^ and then reaches up to the value at 8 × 10^12^ cm^−2^. This awkward behavior can be explained by Figure 4a,b, where mobility initially decrease and then the trend is opposite at higher density, as inversion density decreases in the 40-layer InSe FET. To get physical insight into the mobility degradation, contributions of each scattering mechanism at 5 × 10^11^ cm^−2^ is shown in Figure 6b. AC-, LO-, and HO-limited mobility is severely reduced when NL is less than ~15, while the degradation of remote-phonon as well as Fröhlich-limited mobility begins to decrease about NL ~40, further indicating their major role in determining the electron transport.

**Figure 7:**
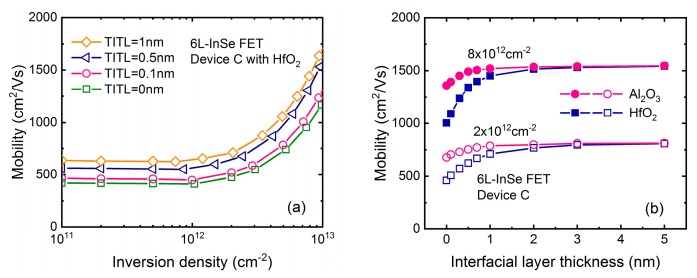
(**a**) Mobility as a function of inversion density featuring different SiO_2_ interfacial layer thickness in InSe FET with dual-gate structure of Figure 1c. (**b**) Mobility as a function of SiO_2_ interfacial layer thickness at different inversion density in both Al_2_O_3_- and HfO_2_-gated InSe FET. EOT = 1 nm of high-*κ* dielectric is used in the simulation.

**Figure 8:**
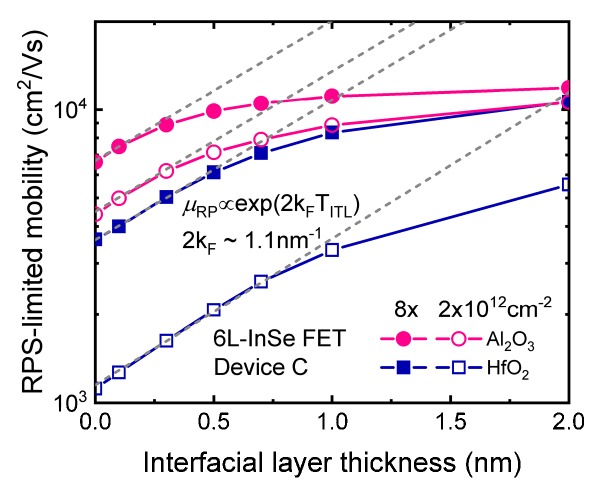
Remote coulomb scattering (RCS)-limited mobility as a function of SiO_2_ interfacial layer thickness corresponding to total mobility in Figure 7b, showing an exponential dependence on T_ITL_ as exp(2*k*_F_T_ITL_) with 2*k*_F_ = 1.1 nm^−1^, represented by the dashed lines.

Inspired by the similar situation for silicon [27,28], it is suggested that an interfacial layer can be introduced between InSe channel and high-*κ* dielectric. To explore the effect of the interfacial layer, Figure 7 shows the calculated mobility in InSe FET with device structure of Figure 1c capped with different high-*κ* dielectric. From Figure 7a, it can be seen that the interfacial layer effectively insulates the channel away from the high-*κ* dielectric, resulting in significant mobility enhancement in the whole range of inversion density due to weaker remote phonon coupling. As interfacial layer thickness is increased, more mobility enhancement is achieved until T_ITL_ approaches ~2 nm, when remote phonon from high-*κ* dielectric is totally separated, as shown in Figure 7b. Besides, a thin interfacial layer is more effective in HfO_2_ dielectric compared with Al_2_O_3_ dielectric. Figure 8 shows the corresponding remote coulomb scattering (RCS)-limited mobility as a function of SiO_2_ interfacial layer thickness, showing an exponential dependence on T_ITL_ as *μ*_RPS_∝exp(2*k*_F_T_ITL_), whatever the inversion density or high-*κ* dielectric is, with Fermi wavelength 2*k*_F_ = 1.1 nm^−1^. This is in agreement with theoretical predictions for a remote scattering mechanism [20,28].

## 4. Conclusions

Based on the self-consistent Poisson and Schrödinger equations and the Kubo–Greenwood formula, remote phonons arising from both the SiO_2_ substrate and high-*κ* dielectrics in InSe FETs are comprehensively studied, together with the intrinsic scatterings by AC phonons, OP phonons, HO phonons, and Fröhlich interaction. It is observed that remote phonons and Fröhlich interaction plays a comparatively major role in determining the electron transport in InSe. Mobility is more severely degraded by remote phonon of HfO_2_ dielectric than Al_2_O_3_ and SiO_2_ dielectric, which can be effectively insulated by introducing a SiO_2_ interfacial layer between the high-*κ* dielectric and InSe. Due to its smaller in-plane and quantization effective masses, mobility begins to increase at higher densities as carriers degenerate, and mobility degradation with reduced layer number is much stronger in InSe compared with MoS_2_.

## Figures and Tables

**Table 1:** Parameters for the polar phonons in some high-*κ* materials [20]

Quantity	SiO_2_	Al_2_O_3_	HfO_2_
*ε*_0_	3.90	12.53	22.00
*ε_int_*	3.05	7.27	6.58
*ε*_∞_	2.50	3.20	5.03
*ћω*_*TO*1_	55.60	48.18	12.40
*ћω*_*TO*2_	138.10	71.41	48.35

*ε*_0_: Static (low-frequency) dielectric constant; *ε_int_*: dielectric constant at an intermediate frequency; *ε*_∞_: high-frequency dielectric constant; *ћω*_*TO*1_, *ћω*_*TO*2_: frequencies of the two polar phonons.
